# Epidemiology of Pediatric Transports and First Aid in a German Municipal Emergency Medical Services (EMS) System: A Cohort Study

**DOI:** 10.1155/emmi/8184007

**Published:** 2025-04-14

**Authors:** Katharina Garrelfs, Benjamin Kuehne, Jochen Hinkelbein, Ralf Blomeyer, Frank Eifinger

**Affiliations:** ^1^Department of Pediatrics, Division of Neonatology and Pediatric Intensive Care, Faculty of Medicine and University Hospital Cologne, University of Cologne, Cologne, Germany; ^2^Department of Anesthesiology, Intensive Care Medicine, and Emergency Medicine, Johannes Wesling Klinikum Minden, Ruhr University Bochum, Bochum, Germany; ^3^Fire Department, Emergency Medical Service, Cologne, Germany

## Abstract

**Background:** Pediatric emergencies remain a significant challenge for emergency services. The study aimed to retrospectively analyze invasive measures and medication administered during prehospital care. The analysis focused on invasive procedures (e.g., tracheal intubation and vascular access) performed on pediatric patients (aged 1 month to 12 years) admitted via the Central Emergency Department (ED) or directly to the University Pediatric Intensive Care Unit (PICU) of the University Hospital of Cologne. These findings provide insights into quality assurance and improvement of prehospital care and invasive emergency techniques in pediatrics.

**Methods:** Emergency protocols were evaluated, including parameters such as the Glasgow Coma Scale (GCS) and National Advisory Committee for Aeronautics (NACA) score. Patients were categorized based on diagnosis, medication administration, and invasive emergency techniques.

**Results:** A total of 373 patients were admitted to the ED, and 237 patients were admitted to the PICU between 01/2015 and 05/2020. Sedation was at similar in both groups, while catecholamines were more frequently used in the PICU group. Invasive procedures, such as tracheal intubation, were rare (PICU: 9.5%; ED: 5.8%; *p*=0.093). Peripheral venous access was performed in 33.7% of PICU cases and 51.2% of ED cases, whereas central venous access was almost never performed. 19 children admitted to the PICU died compared to one in the ED (*p* < 0.001).

**Conclusion:** Invasive procedures are rarely performed during prehospital care for pediatric patients. Trauma cases predominated in the ED group (99.2%), whereas the PICU group exhibited greater diagnostic variability, including trauma and internal emergencies. This study identified significant gaps in medical documentation. Training for paramedics and emergency health workers should prioritize airway management, including supraglottic airway (SGA) devices, thoracic drainage, and vascular access techniques such as peripheral intravenous (PIV) and intraosseous (IO) access. Additionally, efforts to improve medical documentation should be emphasized to enhance pediatric emergency care.

## 1. Introduction

Pediatric emergencies continue to pose significant challenges for emergency services. Although techniques such as tracheal intubation and cardiopulmonary resuscitation (CPR) may resemble those used for adults, understanding the unique anatomical and physiological characteristics of children is essential for effective pediatric emergency management [1, 2]. Furthermore, the wide range of pediatric diseases and the additional complexity of managing communicative and emotional aspects make prehospital care even more challenging [3]. These factors can complicate critical rescue techniques such as tracheal intubation or other invasive procedures.

While all children were first treated by paramedics alone or EMS physicians (without further intensified training in pediatric patients), it is crucial to tailor interventions to the specific needs of pediatric patients [4]. Although the spectrum of emergencies in children may overlap with that of adults, the underlying causes often differ: According to Zwingmann et al. [5], children are particularly vulnerable as road users due to slower reaction times and lack of experience, which predispose them to more severe injuries [6]. While polytrauma is less frequent in children, their risk of life-threatening injuries from multiple traumas is significantly higher. Airway management remains the top priority for all injured patients, as outlined in the German S1 guideline for airway management [7], following the ABCDE scheme. However, establishing vascular access in neonates, children, and adolescents is particularly challenging, and maintaining reliable function is not always guaranteed [8–11].

To enhance the safety of the young patients, healthcare providers must adhere to safety principles and possess specialized knowledge [2, 12]. Despite the recognized importance of accurate monitoring and medication in pediatric emergencies, studies have shown that prehospital care for children—particularly in terms of monitoring and assessment—is less frequent and comprehensive compared to adults [13].

Research highlights that the prehospital management of pediatric patients requires more extensive training compared to adults [14, 15]. Resources for pediatric emergency care, both prehospital and in-hospital, vary significantly depending on logistical factors, infrastructure, and the availability of specialized staff [2]. Larger hospitals with multidisciplinary teams are better equipped to manage pediatric polytrauma cases compared to smaller facilities [16]. Furthermore, effective prehospital care requires early recognition of whether a child needs admission to the Pediatric Intensive Care Unit (PICU) [17, 18]. Ensuring proper pediatric emergency care depends on adequate organization, appropriate equipment, and correct medication [19].

Cologne, a city with 1.1 million inhabitants and the fourth largest city in Germany, presents a unique setting for this study. The University Hospital of Cologne is divided into multiple areas, with the general emergency department (ED) located in a separate building from the PICU. This organizational structure necessitates that paramedics and emergency coordinators decide in advance where to transfer a critically ill child.

This retrospective study aimed to evaluate and analyze invasive measures in prehospital pediatric emergency care at a university hospital providing maximum care.

Additionally, the study assessed the severity and distribution of the clinical conditions, primary in-hospital care pathways, patients' outcomes, and the quality of medical documentation. This work highlights several strategies for ensuring the quality and reliability of invasive pediatric emergency procedures at this facility, with the potential for comparisons with other institutions.

## 2. Methods

### 2.1. Study Design and Setting

This retrospective cohort study was conducted at the University Hospital of Cologne. The study was approved by the local ethics committee of the medical faculty of the University of Cologne (no. 20-1325). This article follows the current Strengthening the Reporting of Observational Studies in Epidemiology (STROBE) guidelines for reporting observational studies (Table S1 in the Supporting Appendix).

### 2.2. Selection of Participants

**Figure 1:**
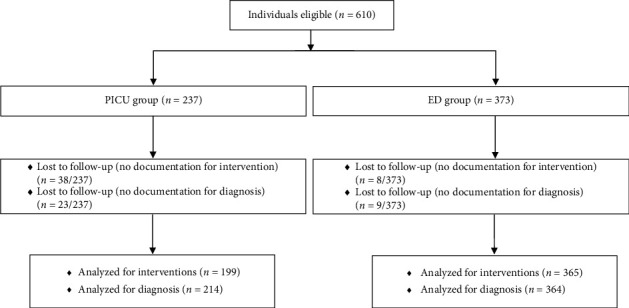
Flow diagram.

Individuals ≤ 12 years old who were admitted either via the shock room of the Central ED or directly to the PICU at the University Hospital of Cologne were eligible for this study (Figure 1). Neonates admitted from the delivery room or following neonatal resuscitation were excluded.

### 2.3. Measures/Outcomes

All study data were collected from patients' medical records and anonymized using the shock room register of the Central ED until discharge from the hospital. The primary outcome was the evaluation of invasive measures performed (e.g. tracheal intubation, types of vascular access, and thoracostomy) in pediatric patients admitted to the ED or the PICU via emergency services.

Further prescribed secondary outcomes included the type of emergency service utilized (paramedic, EMS physician, and EMS helicopter), the Glasgow Coma Scale (GCS) [20], and the National Advisory Committee for Aeronautics (NACA) score [21]. Patients were further categorized based on diagnosis, medication administration, and the use of invasive emergency techniques, including oxygen therapy, tracheal intubation, peripheral venous access, intraosseous (IO) access, central venous access, arterial line placement, or thoracostomy. EMS physicians and/or paramedics performed all invasive procedures. Mortality and hospital length of stay (LOS) were also recorded. For the ED group, the subsequent location of care (intensive care unit, regular ward, or discharge home) was noted. The abbreviated injury scale (AIS) and the injury severity score (ISS) were also determined for the ED group.

### 2.4. Statistical Analysis

Statistical analysis was performed using IBM SPSS Statistics 29.0.0.0 (241) for MacOS Monterey, Version 12.7.2 (*IBM Corp, New York, USA*). Variables are presented as median (IQR), mean ± standard deviation, or absolute and relative frequencies. Differences between groups were compared using the Mann–Whitney U-test (ordinal outcomes) or the chi-square test (nominal outcomes). A two-sided *p* value < 0.05 was considered as statistically significant.

## 3. Results

**Figure 2:**
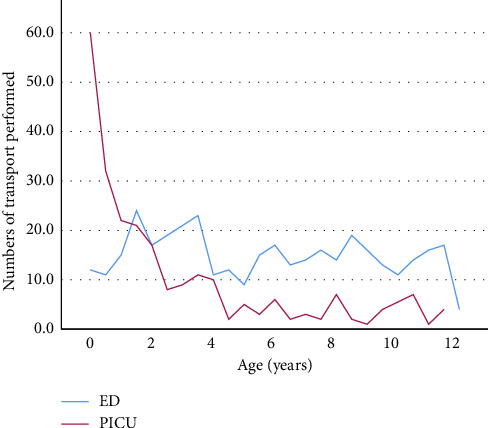
Age distribution of the children studied.

A total of 610 individuals were retrospectively studied between January 2015 and June 2020; 237 individuals were admitted directly to the PICU, and 373 individuals were admitted to the ED. There were 108 (45.6%) females in the PICU, and 146 (39.1%) females in the ED patients, respectively. Patients in the PICU group were significantly younger with a median [IQR] age of 1.58 years [0.5–3.96], compared to a median age of 5.9 years [2.8–8.84] in the ED group (*p* < 0.001, Table 1, Figure 2).

Any invasive or noninvasive procedure was recorded in 92.4% of patients in the PICU group and in 73.4% of patients in the ED group, respectively (*p* < 0.001, Table 2).

The PICU group had a significantly higher incidence of GCS < 9 at admission compared to the ED group with 43.8% versus 5.7% (*p* < 0.001, Table 3). There were significantly more patients with NACA score VI in the PICU group compared to the ED group (12.9% vs. 0.4%, *p* < 0.001), while lower NACA scores of II-IV were significantly more common in the ED group (Table 4). Differences can be seen through all NACA scores; for example, in the PICU Group, 24.7% have a NACA score of III and 41.1% in the ED group. However, there are a large number of undocumented scores for the GCS (62.4% of the PICU and 14.7% of the ED patients) and the NACA (60.8% in the PICU group and 39.7% in the ED group).

Of the 373 children admitted to the ED, 99.2% were trauma patients, whereas a wide variety of pediatric emergencies were seen in the PICU (Table 5: neurological emergencies 30.4%, infections or sepsis 20.6%, and AB-problems 19.2%).

In both groups, more than half of the children receive no medication at all (Table 6: PICU: 65.7%; ED: 69.0%). Sedation and narcotics were administered in 28.8% of the PICU group and in 30.7% of the ED group (*p*=0.582). Catecholamines were administered in 8.6% of the PICU patients and only 1.4% of the ED patients (*p* < 0.001).

Of the 237 patients admitted directly to the PICU, 19 died. Of the 373 patients admitted to the ED, 2 died during this period due to fatal injuries. The PICU patients had a variety of causes including hypoxemia (*n* = 4), infection/sepsis (*n* = 5), resuscitation (*n* = 5), and respiratory failure (*n* = 5). The two patients in the ED group suffered from fatal injuries. AIS scores less or equal to two were 90.1% (Table 7), and ISS scores below 16 were recorded in 89.3% of all ED patients (Table 8).

In addition, in the ED group, half of the patients initially admitted were discharged home (44.2%). A third (31.1%) of the patients were sent to the regular ward, and a few of this group were sent to the PICU (8.8%), operating room (8%), and IMC (2.1%). And we also found a group where the further treatment was not documented (2.7%). This is reflected by the LOS which shows a median [IQR] of 20.3 days [3–9] in the PICU group and 2 days [1–3] in the ER group, respectively (Table 9).

## 4. Discussion

In this 5-year retrospective cohort study of all pediatric patients admitted either via the ED shock room or directly to the PICU, we found that the majority of pediatric patients treated in the ED had a traumatic medical condition and were older, whereas patients admitted directly to the PICU were significantly younger, significantly sicker with higher NACA and lower GCS scores, and had a broader spectrum of nontraumatic illnesses, including primarily neurologic, infectious, and respiratory emergencies.

While our study data suggest an over-triage of polytraumatic patients who were scored in mean with the GCS 14 or above, patients admitted directly to the PICU were significantly under-triaged, as indicated by their substantially lower GCS. The GCS and the NACA score have been used to assess the severity of consciousness and trauma. There is an ongoing debate about the reliability of the GCS score in pediatric populations, particularly given developmental differences in children that may affect their ability to communicate or interact during scoring [22, 23]. Some researchers argue that an age-adjusted GCS score might provide greater accuracy in identifying low- or high-risk children requiring tailored treatments [24]. Notably, the NACA score has demonstrated a stronger correlation with mortality prediction in pediatric trauma cases than the GCS [24].

The modified GCS score may significantly impact outcomes for children admitted to the PICU. One critical objective of utilizing scoring systems such as the NACA and GCS is to guide decisions regarding airway management, including whether tracheal intubation is necessary.

In our study, however, only a small proportion of children underwent prehospital tracheal intubation, a finding consistent with a recent national survey of pediatric emergencies in helicopter emergency services [25]. The relatively low frequency of pediatric prehospital tracheal intubation underscores the necessity for emergency teams to be competent in pediatric airway management. Failure rates for prehospital tracheal intubation in children have been reported to be up to 3.5 times higher than in adults [26, 27]. Therefore, ongoing training in tracheal intubation techniques, including the use of supraglottic airway (SGA) devices, is critical to improve outcomes.

Prehospital invasive procedures, such as central venous catheter (CVC) insertion (*n* = 0), IO vascular access (*n* = 21), or thoracic drainage (*n* = 0), were rare in our study population. In contrast, peripheral intravenous (PIV) vascular access was documented in only 33.7% of PICU patients and 51.2% of ED patients, despite their potentially high risk of morbidity and need for medical intervention. This low rate of PIV access is noteworthy and may reflect difficulties in establishing vascular access in pediatric patients. Myers et al. [28] report that prehospital PIV vascular access is uncommon in children, with success rates strongly correlated to the patient age. Although PIV attempts were not documented in our dataset, it is possible that unsuccessful attempts were made in a larger number of patients.

IO vascular access (*n* = 21; 3.7%) is a well-documented alternative to PIV in pediatric emergencies, particularly when PIV cannot be established properly [29, 30]. Although IO access is described as a reliable and potentially life-saving technique [31], its usage remains limited in pediatric settings [32, 33]. In our cohort, IO was performed in only 7.5% of PICU patients and 1.6% of ED patients, highlighting the need for more widespread adoption of this technique in emergencies. A potential contributing factor may be the limited availability of appropriate IO devices for infants and neonates, as noted by Fuchs et al. [10]. However, high failure rates of > 50% for IO access in pediatric prehospital emergencies, as reported by Harcke et al. [34], may also deter its use. In cases where IO access is challenging, intrabuccal or intranasal medication administration could serve as viable alternatives [35].

Another striking finding in our study was that 65.7% of PICU patients and 69.0% of ED patients received no medical treatment. On the one hand, this could reflect the over-triage of polytraumatized patients in the ED group. On the other hand, the low use of medical treatment in the PICU group, despite the prevalence of infections and cardiovascular and respiratory conditions, is unexpected. This finding aligns with prior reports indicating that prehospital pediatric emergencies are rarely life-threatening and thus often do not necessitate extensive pharmacological intervention [36].

Additionally, pain assessment and management appear to be underemphasized in pediatric trauma care. Prior studies have shown low rates of pain score documentation and opioid administration in injured children [35, 37]. The low frequency of medical interventions in our cohort may therefore reflect challenges in vascular access, insufficient pain assessment, or under-treatment of other manageable conditions.

## 5. Limitations

Our study has several limitations. First, this was a single-center study conducted in a facility with a challenging infrastructure: the ED and the PICU are located in two separate buildings. The ED is equipped with advanced medical imaging capabilities (CT and MRI) and provides access to operating rooms with various surgical specialties, whereas the PICU building lacks these resources. As a result, most trauma patients, regardless of severity, are initially admitted to the ED, while nontrauma patients are more often admitted directly to the PICU. This workflow discrepancy may explain why the overall outcomes in the ED group are favorable, as reflected by higher GCS scores compared to the PICU group.

A second limitation of our study is the lack of a specialized department for burn injuries in our hospital, which necessitated the direct transfer of burn patients to another specialized center. Another important limitation is the incomplete or inconsistent medical documentation, which is a well-documented issue in pediatric emergency care [38], particularly in neonates and infants [39]. Although the implementation of MIND 4.0 documentation standards has been required for years by the German Interdisciplinary Association for Intensive and Emergency Medicine (DIVI) [40, 41], compliance has been inconsistent. It remains unclear whether vital signs and pain scores were recorded but not documented or if they were not assessed at all. This documentation gap could partly explain the observed low rates of medical treatment.

Furthermore, prehospital documentation in our region and much of Germany remains largely paper-based and hand-written [42], which may hinder the completeness and accuracy of records. The digitization of medical documentation, as suggested in other studies, could enhance the quality of record-keeping, improve process efficiency, and provide more reliable data for future research [43].

While the limitations described above may introduce bias into our results, they also restrict the generalizability of our findings to other settings.

## 6. Conclusion

To summarize, life-threatening pediatric emergencies are relatively rare events [44]. The wide variety of diseases or injuries underscores the challenges faced by paramedical teams in responding appropriately to these situations. Given that invasive procedures such as CVCs or arterial lines are infrequently performed, training programs for paramedics and emergency health workers should prioritize core competencies such as pediatric airway management, including chest tube placement, the use of SGA devices and tracheal intubation, and PIV and IO vascular access techniques. In addition, enhancing the quality and completeness of medical documentation should be a key focus in the management of pediatric emergencies [45–47]. Poor documentation not only impacts clinical care but also limits the potential for research and quality improvement in this field. Further studies are needed to address this critical issue and to optimize the management of pediatric emergencies in both prehospital and hospital settings.

## Figures and Tables

**Table 1:** Patient's characteristics.

	PICU group (*n* = 237)	ED group (*n* = 373)	*p* value
*Sex*			
• Male, no. (%)	129 (54.4)	227 (60.9)	0.117^b^
• Female, no. (%)	108 (45.6)	146 (39.1)	0.117^b^
Age, median [IQR], yrs	1.58 [0.5–3.96]	5.9 [2.8–8.84]	<0.001^b^

*Transport*			
• Not documented	56 (23.6)	4 (1.1)	<0.001^a^
• Paramedic alone, no. (%)	33 (18.2)	99 (26.8)	0.03^a^
• Paramedic, emergency physician, no. (%)	100 (55.2)	221 (59.9)	0.351^a^
• Paramedic, emergency physician, helicopter, no. (%)	3 (1.7)	43 (11.7)	<0.001^a^
• Other, no. (%)	44 (24.3)	7 (1.9)	<0.001^a^

*Note:* yrs = years.

Abbreviation: IQR = interquartile range.

^a^Pearson's Chi^2^-test.

^b^Mann–Whitney U-test.

**Table 2:** Prehospital invasive procedures, multiple mentions possible.

Procedures	PICU group (*n* = 237)	ED group (*n* = 373)	*p* value
Not documented, no. (%)	38 (16)	8 (2.1)	<0.001^a^
No procedures, no. (%)	15 (7.5)	97 (26.6)	<0.001^a^
Oxygen, no. (%)	83 (41.7)	54 (14.8)	<0.001^a^
Tracheal intubation, no. (%)	19 (9.5)	21 (5.8)	0.093^a^
Thoracic drainage, no. (%)	0	0	n.c.
Peripheral intravenous vascular access, no. (%)	67 (33.7)	187 (51.2)	<0.001^a^
Intraosseous vascular access, no. (%)	15 (7.5)	6 (1.6)	<0.001^a^
Central venous access, no. (%)	0	0	n.c.
Arterial line, no. (%)	0	0	n.c.

Abbreviation: n.c. = not calculable.

^a^Pearson's Chi^2^-test.

**Table 3:** GCS at initial presentation.

GCS	PICU group (*n* = 237)	ED group (*n* = 373)	*p* value
Not documented, no. (%)	148 (62.4)	55 (14.7)	<0.001^a^
3–5, no. (%)	22 (24.7)	11 (3.5)	<0.001^a^
6–8, no. (%)	17 (19.1)	7 (2.2)	<0.001^a^
< 9, no. (%)	39 (43.8)	18 (5.7)	<0.001^a^
9–13, no. (%)	23 (25.8)	31 (9.7)	<0.001^a^
14-15, no. (%)	27 (30.3)	273 (85.8)	<0.001^a^

Abbreviation: GCS = Glasgow coma scale.

^a^Pearson's Chi^2^-test.

**Table 4:** NACA score at admission.

NACA score	PICU group (*n* = 237)	ER group (*n* = 373)	*p* value
Not documented, no. (%)	144 (60.8)	149 (39.7)	<0.001^a^
I, no. (%)	0	1 (0.4)	0.519^a^
II, no. (%)	4 (4.3)	26 (11.6)	0.043^a^
III, no. (%)	23 (24.7)	92 (41.1)	0.006^a^
IV, no. (%)	34 (36.6)	91 (40.6)	0.5^a^
V, no. (%)	19 (20.4)	13 (5.8)	<0.001^a^
VI, no. (%)	12 (12.9)	1 (0.4)	<0.001^a^
VII, no. (%)	1 (1.1)	0	0.12^a^

^a^Pearson's Chi^2^-test.

**Table 5:** Diagnoses of the patients.

Diagnoses	PICU (*n* = 237)	ED (*n* = 373)	*p* value
Not documented, no. (%)	23 (9.7)	9 (2.4)	<0.001^a^
Trauma, no. (%)	13 (6.1)	361 (99.2)	<0.001^a^
Neurological emergencies, no. (%)	65 (30.4)	3 (0.8)	<0.001^a^
Infection/sepsis, no. (%)	44 (20.6)	0	<0.001^a^
AB-problems, no. (%)	41 (19.2)	0	<0.001^a^
Intoxication, no. (%)	2 (0.9)	0	0.065^a^
Cardiovascular emergencies, no. (%)	17 (7.8)	0	<0.001^a^
Internal emergencies, no. (%)	5 (2.3)	0	0.003^a^
Infant emergencies, no. (%)	11 (5.1)	0	<0.001^a^
Metabolically emergencies, no. (%)	9 (4.2)	0	<0.001^a^
Others, no. (%)	7 (3.3)	0	<0.001^a^

Abbreviation: AB = airway breathing.

^a^Pearson's Chi^2^-test.

**Table 6:** Medication, multiple mentions possible.

Medication (multiple mentions possible)	PICU (*n* = 237)	ED (*n* = 373)	*p* value
Not documented, no. (%)	39 (16.5)	15 (4)	<0.001^a^
No medication, no. (%)	130 (65.7)	247 (69)	0.334^a^
Sedation and narcotics, no. (%)	57 (28.8)	110 (30.7)	0.582^a^
Catecholamines, no. (%)	17 (8.6)	5 (1.4)	<0.001^a^
Antiepileptic drugs/psychotropic drugs, no. (%)	5 (2.5)	2 (0.6)	0.048^a^
Antipyretics, no. (%)	0	0	n.c.
Others, no. (%)	6 (3)	6 (1.7)	0.301^a^

*Note:* Sedation and narcotics: midazolam, lorazepam, diazepam, fentanyl, sufentanyl, piritiramide, morphine, chloral hydrate, ketamine, propofol, thiopental, and etomidate. Catecholamines: acrine, atropine, arterenol, adrenaline, and milrinone. Antiepileptic drugs/psychotropic drugs: Haloperidol, ethosuximide, phenobarbital, levetiracetam, and valproic acid. Antipyretics: metamizole and acetaminophen. Others: succinylcholine, atracurium, rocuronium, ipratropium bromide, amiodarone, and ilomedin.

Abbreviation: n.c. = not calculable.

^a^Pearson's Chi^2^-test.

**Table 7:** AIS head score.

AIS head score	ER group (*n* = 373)
Not documented, no. (%)	1 (0.3)
0 (no injury), no. (%)	270 (72.4)
I (moderate), no. (%)	46 (12.3)
II (minor), no. (%)	20 (5.4)
III (serious), no. (%)	18 (4.8)
IV (severe), no. (%)	13 (3.5)
V (critical), no. (%)	3 (0.8)
VI (fatal), no. (%)	2 (0.5)

Abbreviation: AIS = abbreviated injury scale.

**Table 8:** ISS.

ISS (score)	ER group (*n* = 373)
Not documented, no. (%)	2
0, no. (%)	20
1, no. (%)	16
2, no. (%)	26
3, no. (%)	22
4, no. (%)	120
5, no. (%)	62
6, no. (%)	12
8, no. (%)	28
9, no. (%)	18
10, no. (%)	2
13, no. (%)	7
16, no. (%)	2
17, no. (%)	1
18, no. (%)	4
20, no. (%)	1
22, no. (%)	5
25, no. (%)	7
27, no. (%)	4
29, no. (%)	1
34, no. (%)	5
41, no. (%)	2
50, no. (%)	1
66, no. (%)	3
75, no. (%)	2

Abbreviation: ISS = injury severity score.

**Table 9:** Hospital length of stay.

Hospital length of stay (LOS)	PICU group (*n* = 237)	ED group (*n* = 373)	*p* value
LOS, median [IQR], d	20.3 [3–9]	2 [1–3]	<0.001^a^

Abbreviations: d = days, IQR = interquartile range.

^a^Mann–Whitney U-test.

## Data Availability

All data analyzed during this study are included in this published article. Data and materials are accessible and can be shared when necessary.

## Supporting Information

STROBE-checklist.
